# Recent Advances on Main Active Ingredients, Pharmacological Activities of *Rosa roxbughii* and Its Development and Utilization

**DOI:** 10.3390/foods12051051

**Published:** 2023-03-01

**Authors:** Li Wang, Tingting Wei, Li Zheng, Fangfang Jiang, Wentao Ma, Min Lu, Xiaomao Wu, Huaming An

**Affiliations:** 1 Institute of Crop Protection, Guizhou University, Guiyang 550025, China; 2 Agriculture and Rural Bureau of Majiang County, Majiang 557600, China; 3 Guizhou Fruit Tree Engineering Research Center, Guiyang 550025, China

**Keywords:** *R. roxburghii*, nutritional composition, active ingredient, pharmacological activity, development, utilization

## Abstract

*Rosa roxburghii* tratt (*R. roxburghii*) is an important plant resource that is widely distributed in the southwest of China and favored by consumers due to its high nutritional value and healthy functions. Meanwhile, it is a traditional edible and medicinal plant in China. With the deepening research of *R. roxburghii*, more and more bioactive components and its health care and medicinal value have been discovered and developed in recent years. This review summarizes and discusses the recent advances on main active ingredients such as vitamin, protein, amino acid, superoxide dismutase, polysaccharide, polyphenol, flavonoid, triterpenoid and mineral, and pharmacological activities including antioxidant activity, immunomodulatory activity, anti-tumor activity, glucose and lipid metabolism regulation, anti-radiation effect, detoxification effect, and viscera protection of *R. roxbughii,* as well as its development and utilization. The research status and existing problems of *R. roxburghii* development and quality control are also briefly introduced. This review ends with some suggestions on the perspectives and directions for future research and potential applications of *R. roxbughii*.

## 1. Introduction

**Figure 1:**
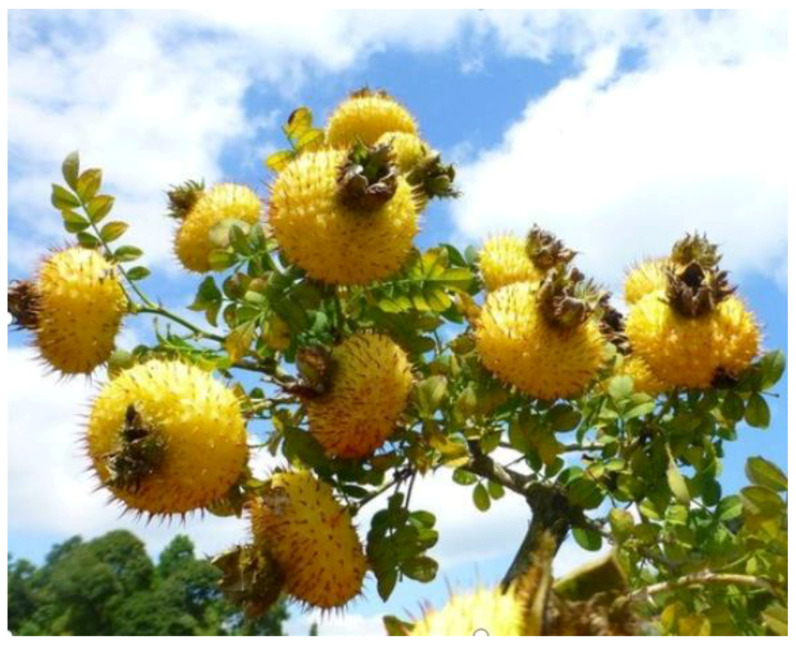
The plant of *R. roxburghii*.

*Rosa roxburghii* tratt (*R. roxburghii*) is a perennial wild deciduous shrub in the Rosaceae family, with a slightly sour state and astringent flavor, strong aroma and crisp texture. It is the third generation (3G) of premium fruit with homology of medicine and food in China. It is suitable for growing in the alpine and hilly areas of temperate and subtropical regions at an altitude of 500 m to 1500 m, especially in the areas with large temperature differences and some cold areas. *R. roxburghii* is an important resource of medicinal and edible origin, which is mainly distributed in southwestern China, especially in Guizhou Province. At present, *R. roxburghii* has been listed as one of the twelve characteristic and advantageous industries in Guizhou Province as a geographical indication protection product. Guizhou Province is the first region to develop and utilize the resources of *R. roxburghii*. It is the only province in China and even the world to carry out large-scale planting and industrial development of *R. roxburghii*. It started with artificial cultivation in the 1980s [1,2]. By the end of 2022, the planting area of *R. roxburghii* in Guizhou Province had reached 140,000 hm^2^, with 300,000 tons of fresh fruits output, realizing an output value of over 15 billion yuan. Although the history of *R. roxburghii* that was planted artificially is short, with the improvement of people’s understanding of the third generation fruit and the in-depth study on nutritional value of *R. roxburghii* fruit, the *R. roxburghii* industry has begun to enter a new stage of variety, cultivation, and comprehensive development. The plant of *R. roxburghii* is shown in Figure 1. This paper reviewed the recent research advances of the nutrients and active ingredients, components and various functional activities of *R. roxburghii*, summarized the development and problems of *R. roxburghii* products, and put forward some suggestions for the future research and development. It is hoped that this review can inspire investigations on *R. roxburghii* as a functional food.

## 2. Traditional Uses

As early as more than 400 years ago, Miao and other ethnic minorities in Guizhou Province had discovered the value of *R. roxburghii* and began to use this resource [3]. The earliest literary record about *R. roxburghii* was written in the “Chronicles of Guizhou” by Tian Wen of the Ming Dynasty in 1640: “*R. roxburghii* resembles pomegranate but is smaller than it. It is an edible wild fruit with a slightly sour astringent flavor, and has the effect of aiding digestion”, and then its medicinal value was firstly record in “The Supplement to the Compendium of Materia Medica”, which was written by Zhao Xuemin in the Qing Dynasty [4]. In addition, it is also recorded in the “Dictionary of Traditional Chinese Medicine” that all the flowers, fruits, leaves, roots, and seeds of *R. roxburghii* can be used as medicine, which have effects of invigorating stomach, aiding digestion, and nourishing, and the root bark has the effect of stopping diarrhea [5]. Meanwhile, *R. roxburghii* has also been used in folk medicine to treat stomach distension, hemorrhoids, dysentery, and other diseases [6]. The most widespread traditional use of *R. roxburghii* is for brewing *R. roxburghii* wine. The origin of *R. roxburghii* wine in Guizhou was recorded very early in the Daoguang Years of the Qing Dynasty. The “Annals of Guiyang Prefecture” of the same year once wrote that “Today, people in Guizhou Province pick *R. roxburghii* fruits, steam them, dry them in the sun, wrap them in cloth, then brew them in a jar full of wine to get *R. roxburghii* wine, which tastes great”. This also reflects the traditional brewing process of *R. roxburghii* wine: Fresh *R. roxburghii* fruits washed and broken → juice → fermentation → clarification and filtration → canned sterilization → *R. roxburghii* wine [7]. By the 1940s, *R. roxburghii* wine in Guizhou province had reached a certain scale, and the *R. roxburghii* industry began to form. In 1951, the earliest *R. roxburghii* processing enterprise “State-owned Qingyan Distillery” (renamed as *Guizhou Huaxi Rosa Roxburghii Wine Distillery* in 1954) was established in *Guizhou Province*, China.

However, the greatest impact of *R. roxburghii* today is the development of functional foods and dietary supplements, which may be widely used in complementary and alternative medicine in the future. Modern science has proved that the nutritional value of 3G fruit is hundreds or even thousands of times of the first two generations of fruit. At the same time, it also plays an important role in admiration, medicinal application, and soil and water conservation, suggesting that planting and developing 3G fruit become a new trend of fruit development in the 21 st century [1]. A large number of scientific studies have shown that *R. roxburghii*, as an emerging 3G fruit, contains in a variety of rich nutrients and active ingredients such as organic acids, superoxide dismutase (SOD), flavonoids, polyphenols, polysaccharides, triterpenoids and so on, which give it biological functions such as promoting gastrointestinal digestion, regulating immune function, delaying aging, anti-cancer, anti-radiation, anti-atherosclerosis, and protecting organs etc [8,9]. Its dual-use attribute of medicine and edible value was recognized, and was included in the *Treasure Book of Ethnic Chinese Medicinal Materials of Guizhou Province* in 2003 [4]. Products developed from *R. roxburghii* are becoming increasingly popular in the consumer market due to their good taste and healthy functions. *R. roxburghii* products have been widely covered in food, medicine, health care products, daily chemical products and other industries, and thus have broad market prospects [10].

## 3. Nutrients and Active Ingredients

Since the 1940s, scientists have conducted a series of studies on the nutritional components of *R. roxburghii*. They found that *R. roxburghii* fruits contained abundant nutrients and active components, such as vitamins, sugars, carbohydrates, organic acids, proteins and amino acids, dietary fibers, trace elements, and other nutrients, active components such as SODs, flavonoids, polyphenols, polysaccharides, triterpenoids, sterols, and glycosides, and volatile components such as nonanal, leaf alcohols, and ethyl oleates [11,12]. In addition, the flowers and leaves of *R. roxburghii* also contained many rich nutrients and active ingredients. Information on the content of important nutrients and active ingredients in different parts of *R. roxburghii* is detailed in Table 1.

### 3.1. Vitamins and Minerals

Vitamins and minerals are essential to the human body, as they play an essential role in a various of basic metabolic pathways that support fundamental cellular functions. In particular, they are involved in energy-yielding metabolism, DNA synthesis, oxygen transport, and neuronal functions, making them essential for brain and muscle function [21]. However, the human body cannot synthesize the vitamins and minerals needed for self-metabolism, and can only obtain them through daily diet. *R. roxburghii* can well meet the needs of the human body of vitamins and minerals, because it is rich in abundant vitamins and trace minerals. The content of vitamin C in *R. roxburghii* fresh fruit is 276.87~3716.19 mg/100 g [14], which is 455~800 times that of vitamin C in apple and 5~40 times that of vitamin C in kiwifruit. In addition, as seen in Table 2, its vitamin A content is 120 times that of apple and 44 times that of mulberry, vitamin E content is 6~7 times that of apple and 12 times that of banana, and carotene content is 145 times that of apple and 96~97 times that of mulberry. The mineral elements in the fruits of *R. roxburghii* are also abundant, among which the contents of Fe, Mn, Zn, B, Cu, P, K, and Ca are higher. In addition to the fruits, the flowers and leaves of *R. roxburghii* are also rich in vitamins and minerals. The content of vitamin C in the petals is 1.7 times of roses (89.57 mg/100 g), and the content of vitamin E is 2.2 times that of *Hemerocallis citrina* (4.79 mg/100 g). The vitamin C content in *R. roxburghii* leaves is 23 times that of *Panax notoginseng* leaves (8.79 mg/100 g). All minerals such as Fe, Mn, Cu, Zn, B, Mo, P, K, Ca, Mg, and Na were found in the flowers and leaves of *R. roxburghii*, and the contents of Fe, Mn, and Zn in the petals were 2.5, 14.6, and 38.7 times that of Fe (4.83 mg/100 g), Mn (0.48 mg/100 g), and Zn (0.18 mg/100 g) in roses, respectively [5,17]. Considering the rich content of vitamins and mineral elements in fresh fruits, flowers, and leaves of *R. roxburghii*, we can develop it into nutritious beverages, scented tea, tea drinks, and other health products to supplement human essentials and vitamins and trace elements under the premise of ensuring that pesticide residues and heavy metal residues are within the safety standards. 

### 3.2. Proteins and Amino Acids

Proteins can be digested and absorbed as amino acids (AAs) and short peptides, and AAs are the cornerstone of proteins, which have structural and metabolic functions in humans and other animals and are very important to human health. Plant protein is one of the important sources of amino acids required for human metabolic activities [23]. The soluble protein content in *R. roxburghii* fruits is 11.62~26.29% [14], and the total free amino acid content is 14.658~57.55 mg/100 g FW, the essential amino acid content is 2.29~12.93 mg/100 g FW, including eight essential amino acids such as threonine and serine and other non-essential amino acids, etc. In addition, there are seven amino acid metabolites found in *R. roxburghii* fruits, such as phosphoserine (P-ser), sarcosine (Sar), α-aminobutyrate (α-ABA), β-alanine (β-Ala), γ-aminobutyrate (GABA), ethanolamine (EOHNH_2_), and hydroxyproline (Hypro). Among them, GABA is an important inhibitory neurotransmitter in the nervous system, which plays an important role in analgesia, anti-anxiety, anti-arrhythmia, neuronutrition and regulation of hormone secretion, and its content in *R. roxburghii* fruits, 27.31~112.17 mg/kg FW, is significantly higher than that of other fruits such as apples, kiwifruits, and cherries. Studies have shown that the content of amino acid in *R. roxburghii* fruits is related to altitude and fruit maturity. The amino acid content of mature fruit is higher than that of half mature fruits, and the content of amino acid in mature fruits of the same variety decreased with the increase of planting altitude [18]. There were 18 protein amino acids and 15 free amino acids detected in the petals of *R. roxburghii*, including eight essential amino acids, and the percentage of essential amino acids to the protein amino acids was as high as 39.62%. The types of amino acids in the leaves of *R. roxburghii* are also abundant, including 18 protein amino acids and 15 free amino acids. The total free amino acid content in the *R. roxburghii* leaves is 6.7 times that of *Eucommia ulmoides* leaves, and its essential amino acid content is 17.7 times that of *E. ulmoides* leaves [5,17]. 

### 3.3. Organic Acids and Vitamin C

Organic acids usually refer to organic compounds containing carboxyl (—COOH) in molecular structure that can neutralize alkali. They are widely distributed in leaves and roots of plants, especially in fruits, and play significant roles in food nutrition, such as antioxidant, sterilization and anti-inflammatory, obesity prevention, regulation of intestinal flora, maintenance of acid-base balance, and resistance strengthening [24]. Common organic acids in fruits include citric acid, malic acid, tartaric acid, acetic acid, succinic acid, oxalic acid, and vitamin C, etc. Vitamin C is an organic acid, also known as ascorbic acid, because the two adjacent enol hydroxyl groups at the second and third positions in its molecule are easily dissociated and release H+, which has the property of acid. At present, the organic acids that have been identified in *R. roxburghii* fruits include vitamin C, lactic acid, malic acid, protocatechuic acid, citric acid, p-coumaric acid, gallic acid, syringic acid, 4-hydroxybenzoic acid, caffeic acid, 9, 12, 15—calendic acid, 9, 12—octadecadienoic acid, tartaric acid, oxalic acid, succinic acid, sorbic acid, linoleic acid, oleic acid, palmitic acid, stearic acid, citric acid, etc. It is found that the content of organic acid components in different parts of *R. roxburghii* was different. The seven main organic acids and their percentages in the total acid content in *R. roxburghii* ripening fruits in descending order were vitamin C (66.8%), malic acid (17.5%), lactic acid (9.9%), tartric acid (2.8%), citric acid (1.7%), oxalic acid (0.7%), and succinic acid (0.6%). The root of *R. roxburghii* mainly contains lactic acid and tartric acid, but almost no oxalic acid, and the high content of tartric acid may be the important reason that *R. roxburghii* root immersed in water had the function of treating diarrhea. The lactic acid content in stems and leaves was higher, especially in *R. roxburghii* leaves, which accounted for more than 45% of the total acid content. The flowers of *R. roxburghii* mainly accumulated succinic acid (accounting for 50% of the total of 7 acids) [25,26,27]. Due to the high proportion of vitamin C in organic acids in *R. roxburghii* fruits and its important medical valuem, most of the current studies on organic acids in *R. roxburghii* focus on vitamin C. These studies mainly include the extraction, purification, quantitative detection, pharmacological activity mechanism of vitamin C in *R. roxburghii*, the change rule of vitamin C content during growth process and the influencing factors of stability during storage and processing.

Researchers have found that the contents of vitamin C in *R. roxburghii* fruits varied greatly depending on the origin, variety, maturity, and altitude, etc. The higher the maturity, the higher the contents of vitamin C, and the lower the area altitude, the higher the contents of it [18]. Moreover, the vitamin C contents of the wild fruit was higher than that of the artificial cultivated fruit [14]. The content of vitamin C in *R. roxburghii* fruits kept increasing during the growth and development, and reached the highest level when mature. The degradation rate of vitamin C in *R. roxburghii fruits* was not obvious at 60 °C, but accelerated when the temperature was higher than 60 °C. When the temperature reached 120C, the vitamin C loss reached 20% [28]. In addition, the loss of water is also an important factor affecting the stability of vitamin C in fresh fruits. The decrease in vitamin C content was significant when the moisture content of *R. roxburghii* fresh fruits was reduced from 85% to 60%, while the decrease in vitamin C content was not significant when the moisture content continued to decrease from 60% [29]. Xiang et al. processed *R. roxburghii* fresh fruits by hot air drying at 60 °C for 30 h and found that the vitamin C content was lost with the increase of temperature and evaporation of water during the drying process. The vitamin C content of dried fruits (7.2 × 10^3^ mg/100 g) was significantly lower than that of freeze-dried fruits (10.8 × 10^3^ mg/100 g), and there was a significant difference (*p* < 0.05). However, the content of vitamin C in dried fruit of *R. roxburghii* was still as high as 7.2% (7.2 × 10^3^ mg/100 g), which did not affect the application quality of *R. roxburghii* [30]. Yan et al. dried the *R. roxburghii* at 37 °C in an oven until the moisture content was <5% (drying time is around 24 h), and compared relative contents of each type of compound in fresh and dried fruits, the result shows that both the total organic acids content and vitamin C content showed no significant difference between these two types of fruits (fold change < 1.5) [31].

The above two studies show that it is very important to find suitable drying and processing methods to reduce the loss of vitamin C and other active ingredients in *R. roxburghii* fruits. Moreover, vitamin C is a key circulating antioxidant and a cofactor of biosynthases synthetase and a gene-regulating enzymes family with anti-inflammatory and immune-supporting effects. It is essential for human beings to prevent scurvy, coronary heart disease, stroke, cancer, and other common and complex diseases. In addition, high doses of intravenous vitamin C have been found to be a low-cost and promising anticancer treatment option [32,33]. Therefore, the research on the qualitative, extraction, purification, quantitative detection, pharmacological activity mechanism, and stability during the processing of vitamin C in *R. roxburghii* is of great significance for the development of functional foods, dietary supplements and alternative medicine of *R. roxburghii* in the future.

### 3.4. Superoxide Dismutase (SOD)

SOD is a kind of metal enzyme that can catalyze the dislocation of superoxide free radical (·O_2_^−^) into hydrogen peroxide (H_2_O_2_) and oxygen (O_2_), which is widely found in animals, plants, and microorganisms. It is the first line of defense for the body to remove active oxygen and help the body resist the damage caused by active oxygen species [34]. A large number of clinical studies have shown that SOD has a positive preventive effect on human cardiovascular diseases, neurodegenerative diseases, and metabolic diseases, including diabetes and its complications and obesity [35]. Due to its powerful activity, it has been widely used in the pharmaceutical industry, food industry, and cosmetics industry [36].

SOD is another active substance with a high content of 33,005~44,650 U/100 g in *R. roxburghii* fruits [13], which is 20~50 times of grape and apple [8] and makes *R. roxburghii* known as “the king of SOD” in the plants. There are many studies on SOD of *R. roxburghii*, mainly involving the extraction and preservation methods of SOD from *R. roxburghii*, and investigations on its enzyme activity, stability, and pharmacological activity, etc. 

Studies have found that the SOD activity in *R. roxburghii* was affected by temperature, pH, the content of vitamin C, the concentration of Cu^2+^ and Zn^2+^, and water content. During the growth of *R. roxburghii* fruits, the SOD activity showed a downward trend, and there was a certain degree of loss with the increase of postharvest storage time. High temperature during processing reduces the SOD activity, while low temperature can protect its activity well [37]. It was found that the SOD activity in *R. roxburghii* fruits was relatively stable at 40~60 °C and a pH value of 7.8~8.0, and the high concentrations of Cu^2+^ and Zn^2+^ had an inhibiting effect on the SOD activity, while 2~6 mmol/L Zn^2+^ had a stabilizing effect on it [28,38,39]. Tan et al. found that the freeze-drying of *R. roxburghii* juice into powder was highly profitable to the preservation of the SOD enzyme [40]. Fu et al. showed that the SOD activity of *R. roxburghii* increased about 1.7 times after sugar pickling [41]. In addition, studies have found that vitamin C has a protective effect on SOD and can significantly slow down the decline of its activity, which is strongly correlated with the content of vitamin C. As mentioned above, the vitamin C content of fresh *R. roxburghii* fruits decreased significantly when the water content decreased from 85% to 60%, however, the SOD activity began to decrease obviously only when the water content decreased from 75% and did not decrease significantly during the process of water content from 85% to 75% [29], which means the decrease of vitamin C content and SOD activity due to water content was not synchronized. It is extremely important for improving the nutritional value of *R. roxburghii* products that to guarantee the vitamin C content and SOD activity during the processing and utilization of *R. roxburghii* fruits. Therefore, how to control the above factors to ensure the vitamin C content and SOD activity of *R. roxburghii* in an optimal content range is an aspect needing attention in the development of high-value *R. roxburghii* products.

### 3.5. Polysaccharides

Polysaccharides are ubiquitous in plants, animals, and microorganisms. Modern pharmacy studies have proved that polysaccharides are non-toxic, green, and safe, which not only provide the main energy needed for human life, but also have a variety of therapeutic effects, including anti-cancer, anti-tumor, anti-diabetes, anti-inflammatory, immune regulation and so on [42]. They are widely added to functional health products and functional foods. It has been confirmed that *R. roxburghii* is rich in polysaccharides, generally ranging from 1.12% to 1.43% [43]. Polysaccharides are a kind of macromolecular substance with extensive biological activities, which are connected by more than ten kinds of monosaccharides through glycosidic bonds, and their activities are related to their structures [44]. Therefore, researchers investigated the biological activity of polysaccharides by isolating them from *R. roxburghii*, and analyzing their molecular weight, monosaccharide composition, proportion, configuration, and the position of the glycosidic bond. There are many extraction methods of *R. roxburghii* polysaccharides at present, such as hot water extraction, alkali extraction, ultrasonic extraction, enzyme extraction, microwave extraction, and so on [45]. The polysaccharide components that have been isolated and studied about their biological activity including RRTP-1, PR-1, PR-2, RTFP, RTFP1-1, RTFP-3, RSPs-40, RSPs-60, RTFP-30, RTFP-50, RTFP-80, RRTFP-2 and RTFP-1, as shown in Table 3. These polysaccharides are mainly composed of mannose (Man), ascorbic acid (AsA), rhamnose (Rha), glucuronic acid (GlcA), galactose (GalA), glucose (Glc), galactose (Gal), arabinose (Ara), xylose (Xyl), fructose (Fru), glucosamine hydrochloride (GluH), fucose (Fuc), glucuronic acid (GluA) and other monosaccharides through different molar ratio and structures, which makes that most of them possess different molecular weight and have strong antioxidant, anti-aging activity and α-glucosidase, α-d-glucosidase and α-amylase inhibitory activity. Some of them have even stronger α-glucosidase inhibitory activity than the hypoglycemic drug acarbose [46,47,48,49,50,51,52,53,54]. These polysaccharide components can be used as a new source of natural antioxidants and hypoglycemic drugs for the development of functional food and dietary supplement products. In addition, studies have found that polysaccharide RRTP-1 has an obvious protective effect on the injury of neural stem cells induced by sodium thiosulfate [55], RRTFP-2 showed the never growth factor (NGF) like neurotrophic activity [56], and the properties (high water solubility and uronic acid content) of the acidic polysaccharide RTFP-3 can facilitate the reduction of AgNO_3_ into Ag nanoparticle composites RP3-AgNPs, which exhibit an excellent antimicrobial ability against Staphylococcus aureus and Escherichia coli compared with RTFP-3, and can provide a green and sustainable strategy for the development of antimicrobial products [57]. In conclusion, the polysaccharide contained in *R. roxburghii* can not only be developed into functional food and medicine, but also into food additives or preservatives, showing the great potential for development and utilization in many fields, and the further study and exploitation of *R. roxburghii* polysaccharide has high theoretical research and practical application value.

### 3.6. Polyphenols and Flavonoids

Polyphenols are natural antioxidants and antimicrobial agents, which are effective against oxidative stress-related diseases. Polyphenolic compounds mainly include flavonoids and non-flavonoids such as phenolic acids, stilbenes, and tannins [58]. Flavonoids are a class of important polyphenols containing γ-pyran group in benzene ring, which have extensive therapeutic activities and can be used as raw materials for antibacterial, anti-inflammatory, immune regulation, heart protection, anti-tumor, anti-aging, and other drugs [59]. *R. roxburghii* is rich in polyphenols and flavonoids. At present, the studies on polyphenols and flavonoids in *R. roxburghii* mainly include the determination, extraction, purification, identification, antioxidant activity analysis, and pharmacological activity analysis, etc. Polyphenols and flavonoids in *R. roxburghii* can be extracted by semi-bionic method, ultrasonic-assisted extraction method, and ethanol reflux extraction method, etc. High-speed countercurrent chromatography was used to separate them with N-hexane:ethyl acetate:ethanol:water (1:20:1:20, *v/v*) as the solvent system [8,60].

Lu et al. found that *R. roxburghii* juice showed the strongest anti-oxidation activity, anti-tumor cell proliferation activity, and the lowest cytotoxicity among the five juices of *R. roxburghii* juice, seabuckthorn juice, lemon juice, blueberry juice, and citrus juice. The determination of total phenol content in these five juices showed that the total phenol content (gallic acid was used as the standard, and the results were expressed as gallic acid equivalent: g GAE/L) in *R. roxburghii* juice (13.06 ± 0.48 g GAE/L) was 40 times that of lemon juice (0.33 GAE/L), 27 times that of citrus juice (0.48 g GAE/L) and 7.2 times that of blueberry juice (1.82 g GAE/L), and the main phenolic compounds detected in *R. roxburghii* juice were gallic acid (5.48 mg/100 mL), rutin (53.61 mg/100 mL), and catechin (68.69 mg/100 mL). The high total phenol, rutin, catechin, and vitamin C contents in *R. roxburghii* juice are important factors for these strong activities of *R. roxburghii* juice [58]. Therefore, polyphenols and flavonoids in *R. roxburghii* have high development and utilization value.

Fan et al. determined 10 phenolic acids and 13 flavonoids in the leaves, petals, and fruits of *R. roxburghii* (Table 4). It can be seen from the Table 4 that the total content of phenolic acids in leaves, petals, and fruits of *R. roxburghii* was 3928.20, 1389.61, and 3356.68 mg/100 g DW respectively, and the total content of flavonoids was 1864.95, 1962.55, and 4956.70 mg/100 g DW, respectively. It is worth noting that the contents of catechins in leaves, petals, and fruits are quite high, with 867.41, 720.7 and 1114.18 mg/100 g, respectively. The tannic acid content in *R. roxburghii* fruits was as high as 2624.98 mg/100 g, accounting for 78.2% of the total phenolic acid in the fruit [15]. While polyphenols in *R. roxburghii* fruits contained tannins, which lead to a certain astringency in the taste of fruits, and the removal of tannins is one of the bases for the taste of *R. roxburghii* products widely loved and accepted by consumers. Research in this area has been rarely conducted, and more in-depth studies are needed to improve the utilization rate and market share of *R. roxburghii* products.

### 3.7. Triterpenoids

Triterpenoids are widely distributed in nature with various types and complex skeleton structures. They have attracted wide attention due to their anti-tumor, anti-virus, antibacterial, anti-inflammatory, and immunoregulatory activities [61]. The total triterpene content of *R. roxburghii* ripening fruit is 22.56–32.32 mg/g DW, which is significantly higher than that of apple, jujube, and other common fruits [18]. Fan et al. detected four triterpenoids including echinacoside, roseoside, rosolic acid, and ursolic acid from the leaves, petals, and fruits of *R. roxburghii,* and the contents of each component in different parts of *R. roxburghii* are shown in Table 5, where that the contents of four triterpenes in leaves were the highest, reaching 1423.64 mg per 100 g of dried leaves, followed by 1055.16 mg in fruits, and the contents of four triterpenes in flowers were relatively low. The total content of echinacoside was the highest among the four triterpene components [15]. In addition to the high content of triterpenes in *R. roxburghii*, its species are also abundant. The components that have so far been identified are polygalacic acid 3-O-β-D-glucopyranoside, 19α-hydroxyasiatic acid-28-O-β-D-glucopyranoside, kajiichigoside F1, 1-hydroxyeuscaphic acid, 2α,19α-dihydroxy-3-oxo-urs-12-en-28-oic acid and isomers, euscaphic acid, pomolic acid and isomers, ursolic acid, 2α, 3β-dihydroxylup-20(29)-en-28-oic acid, 1α, 2β, 3β,19α-tetrahydroxyurs-12-en-28-oic acid, aiiichigeside F1,potentilanoside B, rosamultin, and 2α,3α,19α-trihydroxy-olean-12-en-28-oic acid-28-O-β-D-glucopyranoside [8]. Studies have found that the total triterpene extraction yield of refluxing process was higher than other extraction methods such as boiling and precipitation with ethanol, macro absorption resin, impregnation method, and supercritical fluid extraction, and the extraction rate was as high as 72.4% [62]. Although triterpenoids are very important pharmacologically active substances, and their content is very high in the fruits and leaves of *R. roxburghii*, there are few studies on triterpenoids. It is necessary to further strengthen the research on the extraction, separation, and pharmacological activity of triterpenoids from *R. roxburghii* in the future, so as to fully develop the value of triterpenoids in the prevention and treatment of diseases.

## 4. Pharmacological Activities

With the deepening studies on nutrients and active ingredients of *R. roxburghii* and mechanisms of various active functions in recent years, various pharmacological fuctions of *R. roxburghii* have been gradually discovered. The following is the detailed introduction of the research on several important pharmacological activities of *R. roxburghii*.

### 4.1. Antioxidant Activity

Oxidative stress can cause the body to produce excessive reactive oxygen species, such as DPPH·, ABTS·, ·O_2^−^_, and OH·, which can cause damage to the body and lead to various diseases. Scientific studies have shown that the increase of SOD, glutathione peroxidase (GSH-Px), catalase (CAT), hydroxyproline (HYP), and hyaluronic acid (HA) in the human body can inhibit the oxidation and aging of the body. *R. roxburghii* is rich in vitamin C, SOD, vitamin E, polyphenols, flavonoids, β-carotene, polysaccharides, and other antioxidant substances, which can increase the content of plasma antioxidants, improve the activity of antioxidant enzymes (GSH-Px, CAT and SOD) in vivo, increase the levels of HYP and HA, and reduce the level of ROS in the body. In the meantime, they can improve the antioxidant capacity of plasma low density lipoprotein (LDL), reduce the accumulation of oxidized LDL and the lipid peroxidation product, malondialdehyde (MDA), protect cells from lipid oxidation, and improve the antioxidant and anti-aging ability of the body [63,64,65], regulate the Na^+^ and K^+^ levels of erythrocyte membrane and protect the activity of adenosine triphosphate (ATP), thereby inhibiting the activity of monoamine oxidase (MAO) in the brain and reducing the deoxygenation of MAO [9,66]. In addition, Zhang et al. found that *R. roxburghii* fruit powder can regulate the expression of nuclear factor E2-related factor 2 (Nrf2), B cell lymphoma factor-2 (Bcl-2), and heme oxygenase-1 (HO-1) protein in skeletal muscle tissue of overtraining rats, reduce cell apoptosis, and alleviate the oxidative stress injury of skeletal muscle movement [66].

#### 4.1.1. Antioxidant Activities of Vitamin C, Polyphenols, Flavonoids, SOD, and Triterpenoids

Vitamin C, polyphenols, flavonoids, SOD, and triterpenoids are several important antioxidant substances in *R. roxburghii*, which play an antioxidant role by directly scavenging free radicals, regulating the activity of related enzymes, and chelating metal ions (Fe^3+^) involved in the formation of free radicals. Moreover, the free radical scavenging ability of polyphenols was significantly increased after purification by AB-8 macroporous resin, and the antioxidant activity of flavonoid-refined products was stronger than that of crude flavonoids [67,68]. All the leaves, flowers, roots, and fruits of *R. roxburghii* had strong antioxidant activity. Yan et al. evaluated and compared the constituents and in vitro antioxidant activities of fresh and dried *R. roxburghii* fruits for the first time. A total of 95 compounds, mainly including organic acids, phenols, and flavonoids were identified in fresh and dried fruits by using ultrahigh—performance liquid chromatography—quadrupole—time of flight mass spectrometry. It can be seen from Table 6 that the contents of phenols and acylamide in *R. roxburghii* fruits were significantly increased, while contents of flavonoids, organic acids, and terpenoids reduced after the drying process. However, the scavenging free radical and ferric reducing capacity assays indicated that the dried fruit showed stronger antioxidant activities. The high content of phenols in dried fruits might explain the above-mentioned results [31]. Other studies have also demonstrated the important role of phenols in the antioxidant activity of *R. roxburghii*. Yang et al. investigated and compared the phenolics in free and bound forms of two cultivars of *R. roxburghii* leaves, and their bioactivities, and found that the total amount of free phenols in the leaves of both cultivars was significantly higher than that of bound phenols, and free phenols in both cultivars showed significantly higher antioxidant activity and α-glucosidase inhibitory potency than bound phenols. The characterization and quantitative analysis of phenolic compounds in two leaves showed that the main active components of free phenols were ellagic acid, quercitrin, isoquercitrin, and quinic acid [69]. Tan et al. [19] found that the antioxidant activity of different medicinal parts of *R. roxburghii* was vitamin C > leaves > fruits > roots, which was positively correlated with the content of ellagic acid in different parts, meaning that the higher the content of free (total) ellagic acid was, the stronger the antioxidant activity of the corresponding medicinal parts was. Fan et al. revealed that the scavenging capacity of DPPH· and ABTS· and the reducing capacity of Fe^3+^ in leaves, flowers, and fruits of *R. roxburghii* were in the order of fruits > leaves > flowers, and the scavenging capacity of OH· and ·O_2^−^_ was in the order of fruits > flowers > leaves. The reasons for the above differences were not only related to the differences of the contents of polyphenols, flavonoids, and triterpenes in leaves, flowers, and fruits, but also possibly related to the differences in vitamin C, vitamin E, and SOD contents. The total contents of vitamin C, polyphenols, flavonoids, SOD, and triterpenoids in *R. roxburghii* fruits were much higher than those in flowers and leaves and the synergistic effects of these antioxidants greatly enhanced the antioxidant capacity of *R. roxburghii* fruit extracts [15]. The results of principal component contribution rate analysis show that the contribution of the five active substances to the anti-oxidation ability was in the order of total phenol, vitamin C content > total triterpene content, SOD activity > total flavone content. In addition, it was found that the content of total triterpenoids in old leaves of *R. roxburghii* growing for 100~105 days was the highest, the content of total phenols and total flavonoids in mature leaves of *R. roxburghii* growing for 60~65 days was the highest, and the content of flavonoids in leaves was higher than that in fruits, while the content of vitamin C and SOD activity in mature leaves was significantly lower than that in fruits [70]. Therefore, mature leaves can be used as raw materials for total triterpenoids, polyphenols, and flavonoids in *R. roxburghii*, and the extraction and purification of vitamin C and SOD should be mainly based on fruits.

#### 4.1.2. Antioxidant Activity of Polysaccharides

In recent years, studies on the extraction and activity of *R. roxburghii* polysaccharides have found that many extracted polysaccharide components have scavenging effects on free radicals OH·, DPPH·, ABTS·, and ·O_2^−^_ with different degrees (Table 3). The polysaccharides PR-1 and PR-2 extracted by Wang et al. showed significant DPPH· free radical scavenging activities, and the scavenging ability of PR-1 was equivalent to that of ascorbic acid [46]. The polysaccharides RSPs-40 and RSPs-60 extracted by Chen et al. had a certain scavenging effect on free radicals ABTS· and DPPH·, but both of them were weaker than ascorbic acid [50]. The new water-soluble polysaccharide RRTP1-1 extracted by Chen et al. not only showed strong OH·, ·O_2^−^_, and DPPH· scavenging ability, but also possessed obvious antioxidant activity in vitro [51]. At the dose of 200 mg/kg, it could significantly enhance the activity of antioxidant enzymes (SOD, CAT and GSH), and reduce the levels of lipid peroxidation and MDA in the serum of aging mice induced by D-galactosamine. The three polysaccharide components, RTFP-30, RTFP-50, and RTFP-80, isolated by Wang et al., showed a varying degree of scavenging effect on OH·, ABTS·, and DPPH· at a certain concentration, and scavenging ability of RTFP-50 was the strongest, which may be due to its strong electron or hydrogen atom donor [52]. The free radical scavenging activities of the above polysaccharide components are in direct proportion to the concentration of *R. roxburghii* polysaccharide in a certain concentration range. In addition, Cao et al. found that polysaccharide of *R. roxburghii* could not only increase the content of SOD, CAT and GSH, reduce the level of MDA, but also eliminate fatigue by providing energy substances such as blood sugar and muscle glycogen needed during exercise and reducing excessive metabolism of adverse substances such as lactic acid, lactate dehydrogenase, and creatine kinase [71]. 

### 4.2. Immunomodulatory Activity

The immune system is a barrier to maintain normal body function and can resist foreign matters. The disorders of immune system can lead to tumors, inflammation, infection, and other diseases, which will seriously threaten human life and health. Liu et al. found that *R. roxburghii* freeze-dried powder can reduce the expression of immune inflammatory factors in the kidney of model rats, regulate the immune microenvironment, and improve renal fibrosis in rats [72]. Lu et al. found that polysaccharide of *R. roxburghii* can increase the percentage of phagocytosis of chicken erythrocytes by mouse macrophages, prolong the half hemolysis value of serum, and enhance the non-specific immune function and humoral immune function of mice [73]. The experimental results on the immunity of total triterpenoids from *R. roxburghii* holded by Tian et al. showed that the total triterpenoids of *R. roxburghii* could alleviate the damage of cyclophosphamide (CTX) injection on thymus and spleen, improve the number of immune cells, enhance the anti-oxidative stress ability of mice and enhance the immune function of the body [74]. It could also regulate the activities of acid phosphatase (ACP) and lactate dehydrogenase (LDH) in mice, promote the proliferation of RAW264.7 macrophages in mice, and inhibit the secretion of NO by macrophages induced by lipopolysaccharide (LPS), which indicates it have potential anti-inflammatory and immune activities. In addition, clinical studies have shown that urine arsenic levels of patients with coal-burning arsenism were closely related to their immunosuppression, and *R. roxburghii* preparation could effectively improve the immune function of patients with coal-burning arsenism [75].

### 4.3. Glucose and Lipid Metabolism Regulation

The balance of glucose and lipid metabolism is the basis of the body’s life activities, which plays an important role in the normal physiological function. Glucose and lipid metabolism disorder is an important reason for major chronic metabolic diseases such as diabetes, obesity, hyperlipidemia, and atherosclerosis [76]. Previous animal tests and clinical studies have found that *R. roxburghii* has good effects for regulating glucose and lipid metabolism.

#### 4.3.1. Reducing Blood Glucose Effect

Diabetes is a metabolic disease characterized by hyperglycemia. The most common forms of diabetes are type 1 diabetes caused by the absolute deficiency of insulin, and type 2 diabetes caused by insulin resistance. Obesity is an important environmental factor for type 2 diabetes, and it is on the rise. Diabetic complications affect almost every tissue of the body, which is the main cause of cardiovascular disease and death, blindness, renal failure, and amputation [77]. Recent studies have revealed that polysaccharides, polyphenols, flavonoids, and triterpenoids in *R. roxburghii* have shown certain hypoglycemic activities, and there are mainly two ways that *R. roxburghii* exerts the hypoglycemic activity.

The first way that *R. roxburghii* reduces the blood sugar levels in the body by inhibiting the activities of α-glucosidase, α-amylase, and α-D-glucosidase, then reduces the absorption rate of blood glucose, and achieves the purpose of preventing diabetes and alleviating the symptoms of diabetes. For example, both the polysaccharide components PR-1 and PR-2 extracted by Wang et al. showed a certain α-d-glucosidase inhibitory activity, and the inhibitory activity of PR-1 was stronger than PR-2, but both of them were weaker than acarbose [46]. RTFP-3 purified by Wang et al. [47], RSPS-40 and RSPS-60 extracted by Chen et al. [50], and RTFP-50, RTFP-80 isolated by Wang et al. [52] all had strong α-glucosidase inhibitory activity, which can reduce the absorption rate of blood glucose and prevent type 2 diabetes mellitus, and the effect of RSPS-40 and RSPS-60 was better than that of acarbose. In addition, RTFP, a polysaccharide component isolated by Wang et al. [49], had strong inhibitory activity against a-glucosidase and a-amylase, and its inhibitory activity was much higher than that of acarbose. Due to the strong activity of RTFP, Wang et al. further explored its potential hypoglycemic mechanism [48]. They found that RTFP could significantly improve the insulin resistance in DB/DB diabetic mice, increase their SOD, GSH, and catalase (CAT) activities in liver tissue, significantly reduce the weight, fat, liver hypertrophy, fasting blood glucose, serum insulin, and blood lipid levels of mice, enhance their glucose tolerance, and significantly improve the symptoms of hyperglycemia and hyperlipidemia. Zhu et al. found that polyphenols and flavonoids in *R. roxburghii*, especially the catechin, kaempferol hexose and rutin of them, had strong inhibitory activity on a-glucosidase, and the catechin, the crude extracts of flavonoid, had a good synergistic effect with acarbose, which had potential application value in reducing the clinical dosage of acarbose [78]. Qin et al. found that the triterpenes of *R. roxburghii* had great α-glucosidase inhibitory activity, which was much stronger than that of acarbose [62].

The other way is to regulate the expression of protease through multiple cellular signaling pathways to exert its hypoglycemic effect. For example, An et al. found that *R. roxburghii* fruit wine could shift glucose transporters 2 and 4 (GLUT2/4) by activating the phospholipid phthalinositol 3-kinase (PI3K) pathway of insulin pathway mediated by serum insulin (INS), promote the absorption and utilization of glucose by cells, thereby reduce the blood glucose level of type 1 diabetic mice [79]. It could also up-regulate the mRNA expression levels of AMP-activated protein kinase α (AMPK), glucose transporter 2 (GLUT2), acetyl-CoA carboxylases alpha (ACACA), fatty acid synthase (FASN) in liver tissue and down-regulate the mRNA expression levels of phosphoenolpyruvate carboxylase (PEPCK), glucose-6-phosphatase (G6Pase), HMG-CoA reductase (HMG-CoA) and cholesterol 7 α -hydroxylase (CYP7A1), significantly reduce the body weight, fat, liver hypertrophy and fasting blood glucose, serum insulin and lipid levels in db/db mice, and effectively alleviate the symptoms of type 2 diabetes [80]. Chen et al. isolated polyphenols-rich *R. roxburghii* extract (RP) from *R. roxburghii* fruits and separated four components (IRP1-4) from RP [81]. They found that both RP and IRP1-4 could regulate the expression of FOXO1 (a downstream protein of the P13K/AKT pathway) and p-GSK3 protein, control liver gluconeogenesis, improve liver glycogen storage insulin resistance, and relieve symptoms of type 2 diabetes by activating the expression of phosphatidylinositol 3-kinase (P13K)/thephosphorylation of protein kinase B (AKT) signaling pathway. It is worth noting that the cellular signal transduction pathway is very complicated, and different pathways often interact with each other. The same polysaccharide can often regulate multiple signal transduction pathways at different levels and at different links at the same time.

In addition, Wang et al. firstly constructed selenium nanoparticles (SeNPs) functionalized with the RTFP-3, the novel polysaccharide extracted from *R. roxburghii* fruits mentioned above, via a facile, single -step, and green in situ synthesis method. They found that the RTFP-3-functionalized SeNPs (RP3-SeNPs) exhibited high dispersibility and stability, and could significantly inhibit the H_2_O_2_-induced apoptosis of INS-1 cells by attenuating oxidative stress and downregulating the expression of uncoupling protein-2 (UCP-2), which demonstrate that RP3-SeNPs may be a promising candidate for the treatment of ROS-mediated diabetes [54].

#### 4.3.2. Reducing Blood Lipid Effect

Hyperlipidemia is a manifestation of abnormal lipid metabolism. Hyperlipidemia related to lipid disorders are considered to be the cause of atherosclerotic cardiovascular disease. It is characterized by an increased levels of plasma lipid, such as total cholesterol (TC), triglyceride (TG), cholesterol ester, very low density lipoprotein cholesterol (VLDL-C), low density lipoprotein cholesterol (LDL-C), free fatty acids, and the reduced levels of high density lipoprotein cholesterol (HDL-C) [82]. Therefore, lowering blood lipids is an effective method to prevent and treat cardiovascular diseases.

Zhang et al. found that flavonoids from *R. roxburghii* could significantly improve the activities of SOD and CAT in the pancreas, markedly reduce the level of MDA and the content of serum glucose, triglyceride and total cholesterol, increase serum insulin levels, and effectively protect the pancreas from oxidative damage of alloxan [83]. Zhou found that the aqueous extract of *R. roxburghii* could significantly reduce the triglyceride and cholesterol values of hyperlipidemia mice (*p* < 0.01), and speculated that its effect might be related to the water-soluble components such as organic acids [84]. Wu et al. found that the hydroalcoholic extract of *R. roxburghii* fruit (HRT) could significantly reduce body weight gain and decreased serum and liver lipid levels in the hyperlipidemicrats by improving the activities of antioxidant enzymes, lipoprotein lipase, hepaticlipase, and regulating the expressions of related mRNA and protein [85]. HPLC-MS analyses showed that the total phenolic acid content in HRT was 88.30%, including phenolic acids such as L-ascorbic acid, kaempferol, catechin, rutin, and isoquercitrin. Many previous studies have shown that phenolic acids are involved in lipid-lowering effects, such as ascorbic acid alleviating alcohol-induced hyperlipidemia, rutin preventing hypertriglyceridemia and inflammation, and green tea catechins effectively preventing obesity and hypercholesterolemia. These results indicate that phenolic acids played an important role in the hypolipidemic effect of hormone replacement therapy. The research of Wang et al. shows that polysaccharide of *R. roxburghii* also showed strong lipid-lowering activity. They compared in vitro binding characteristics of RTFP-30, RTFP-50 and RTFP-80, the three polysaccharide components isolated from *R. roxburghii*, and found that RTFP-30 and RTFP-50 showed strong binding ability to fat, cholesterol and bile acid, which had great potential in preventing obesity and hypercholesterolemia [52].

#### 4.3.3. Intestinal Flora and Lipid Metabolism Regulating

Intestinal flora is closely related to hyperlipidemia, which can regulate cholesterol and lipid metabolism in the host, and regulating intestinal flora and liver fat metabolism is an effective way to prevent diseases related to glucose and lipid metabolism disorders. Ji et al. found that the fermented *R. roxburghii* juice (FRRT) could alleviate hyperlipidemia induced by high fat diet in rats by regulating intestinal flora (prevotella, oscillospira, paraprevotellaceae prevotella and ruminococcus) and related metabolites (amino acid metabolites, bile acid metabolites and lipid metabolites) [86]. The chemical composition of FRRT is 13.49 mg/mL total flavonoids, 23.91 mg/mL total polysaccharides, 35.97 mg/mL total polyphenols, and 7.58 mg/mL vitamin C. It can be seen that polyphenols, polysaccharides, flavonoids, and vitamin C are the main active components of FRRT regulating intestinal flora in *R. roxburghii* juice. Except for lowering blood glucose, the polysaccharide component (RTFP) separated by Wang et al. can also be used as a natural anti-inflammatory agent to reduce chronic obesity-induced colitis [49]. It can significantly decrease gut inflammation and ameliorate the metabolic dysbiosis of intestinal microflora by decreasing the firmicutes/bacteroidetes ratio, reducing the levels of serum D-lactic acid and lipopoly-saccharides, inhibiting the TLR4/NF-κB signaling pathway, increasing the abundance of beneficial bacteria (ruminococcaceae, muribaculaceae, akkermansiaceae, etc.), and decreasing the abundance of pathogenic bacteria significantly [87].

There is a close relationship between inflammation of adipose tissue and obesity. In adipose tissue of obese patients, M1 phenotype macrophages account for the majority; in lean adipose tissue, M2 phenotype macrophages are the majority. Obesity leads to the transformation of adipose tissue macrophages from M2 phenotype to M1 phenotype. M1 phenotype macrophages secrete inflammatory factors, which aggravates adipose tissue inflammation, and eventually leads to obesity complications such as insulin resistance. Sui et al. found that the drug-containing serum of *R. roxburghii* produced in Guizhou could promote the transformation of adipose tissue macrophages from M1 phenotype to M2 phenotype by regulating the expression of related transformation factors and inflammatory factors, so as to achieve the effect of weight loss and inflammation and effectively prevent obesity complications such as insulin resistance [88].

#### 4.3.4. Anti-Atherosclerosis Activity

Atherosclerosis (AS) is a kind of disease that mainly invades the great and middle arteries, thickens and hardens the inner wall of blood vessels, and narrows the lumen. Abnormal lipoprotein metabolism is the main cause of atherosclerosis, and both lipid excess and hyperlipidemia can lead to atherosclerosis. Individuals with high low-density lipoprotein (LDL) level and low high-density lipoprotein (HDL) level in plasma are prone to cardiovascular diseases [89]. Studies have shown that *R. roxburghii* can significantly reduce the contents of TC and triglyceride (TG) in serum, enhance the antioxidant activity of LDL, increase the level of HDL, reduce the damage of lipid metabolism and oxidation to arterial intima, adjust lipid metabolism of hyperlipidemia, and improve SOD activity, so as to prevent atherosclerotic plaques in arterial intima [90,91]. Zhang et al. showed that *R. roxburghii* can also inhibit the formation of foam cells induced by oxidized very low-density lipoprotein (Ox-VLDL) to achieve its anti-atherosclerosis function [92]. In addition, clinical studies have shown that oral administration of *R. roxburghii* juice in patients with cerebral infarction can effectively improve the symptoms of atherosclerosis and reduce the recurrence rate of patients [93]. Some scholars believe that these effects of *R. roxburghii* are closely related to the vitamin C in the fruit, but there is no more in-depth study on the related effective components and their mechanism of action.

### 4.4. Anti-Tumor Activity

With the change of society and human lifestyle, diseases are also gradually changing and developing, including some diseases with unknown causes. Cancer is undoubtedly one of the biggest threats to human health and life. A large number of studies have shown that *R. roxburghii* can block the synthesis of N-nitroso compounds in vivo, induce apoptosis of cancer cells, inhibit the proliferation of various tumor cells, and thus play an anti-cancer role. For instance, the extract of *R. roxburghii* has obvious inhibitory effect on the growth of many human cancer cells, such as human esophageal squamous cell carcinoma CaEs-17, gastric cancer cell SGC-7901, lung cancer A549, human liver cancer SMMC-7721 cells, human CD34+ hematopoietic cells, human leukemia K562 cells, endometrial cancer, human ovarian cell line CoC2, etc [2,94,95,96].

Studies have shown that polysaccharides and triterpenoids are important anti-tumor active ingredients of *R. roxburghii*. Chen et al. found that the crude polysaccharide of *R. roxburghii* could inhibit the proliferation of ovarian cancer cell A2780 by inhibiting the expression of MMP-9 gene related to cancer cell proliferation through a dose-dependent manner [97]. Tang et al. found that polysaccharide of *R. roxburghii* extracted by optimizing microwave assistance had obvious anti-tumor activity and could significantly increase the number of white blood cells, thymus index, and spleen index of S180 tumor mice, which can be used as potential natural sources of functional food additives or anti-tumor drugs [98]. Chen et al. showed that polysaccharides of *R. roxburghii* could inhibit the proliferation of B16 melanoma cells in mice in vitro and in vivo, promote the apoptosis of mouse B16 cells in vitro, and improve the activation function of the immune system, thereby inhibiting tumor formation [99]. On the gene level, polysaccharides of *R. roxburghii* can reduce the expression of anti-apoptotic gene Bcl-2 and increase the expression of pro-apoptotic gene Bax in B16 cells in a dose-dependent manner. Jin et al. isolated and purified a novel polysaccharide RTFP-1 from *R. roxburghii* fruits, and found that RTFP-1 could inhibit the cell proliferation of human hepatocellular carcinoma cells by activating the apoptosis of HepG2 cells through ROS-mediated MAPK, STAT, and p53 apoptotic pathways [53]. Dai et al. found that triterpenoid of *R. roxburghii* had the effect of anti-human endometrial adenocarcinoma in vitro, and the mechanism might be related to its cytotoxicity, induction of cell differentiation, induction of apoptosis, and inhibition of cell proliferation [100]. Huang et al. reported that triterpenoid of *R. roxburghii* had an in vitro anti-proliferation effect on human liver cancer cell SMMC-7721, and its mechanism might induce cell differentiation by down-regulating the expression of bad mRNA, which is not related to inhibiting cell proliferation and inducing cell apoptosis [101].

### 4.5. Anti-Radiation Effect

The radiation protection is an important means to reduce the side effects of radiotherapy. Xu et al. found that flavonoids from *R. roxburghii* could regulate the Bcl-2 (Ca (2+))/Caspase-3/ PARP-1 and PARP-1/AIF apoptosis signaling pathways to reduce cell apoptosis and DNA damage, correct radiation-induced histopathological changes, promote the formation of splenic nodules, resist sperm distortion and protect the thymus, and had a significant anti-radiation effect [102,103]. Polyadenosyl diphosphate polymerase-1 (PARP-1) is the most abundant isomer in the PARP gene family, which is involved in many cellular functions, including DNA repair, post-transcriptional gene expression, regulation of inflammation, and cell death. Mitochondrial reactive oxygen species (ROS) are a DNA damage agent produced by cells due to radiation, which can cause base damage, single or double strand breaks, and stimulate mitotic cell cycle arrest. PARP-1 and ROS are two major participants of radiation injury. Xu et al. found that flavonoids from *R. roxburghii* could control the production of ROS by regulating PARP-1, thereby protecting DNA from radiation damage to play its anti-radiation effect [104].

### 4.6. Detoxification Effect

Studies have shown that heavy metal poisoning by Pb, Mn, Cd, As, Hg, and F can inhibit the antioxidant function and immune function of the body, aggravating the damage of lipid peroxidation to the body serum, liver, kidney, and central nervous system, which in turn leads to skin mucosal lesions, polyneuritis, liver, and kidney function damage. *R. roxburghii* has obvious detoxification and detoxification effects on these heavy metal poisoning, and the mechanism may be related to its vitamin C, SOD, polysaccharide, and trace elements [105]. Studies have shown that *R. roxburghii* can promote the excretion of heavy metals, supplement trace elements in the body, enhance SOD activity in blood, liver, and kidney tissues, and promote the increase of T lymphocytes in the body, significantly improve the immune function and antioxidant capacity of the body, and reduce the lipid peroxide (LPO) and lipid peroxidation degradation product malondialdehyde (MDA) content, thereby lowering the total metal accumulation in blood, liver, and kidney tissues, and reducing the secondary damage caused by heavy metal poisoning [106,107,108,109,110,111]. The clinical trials have shown that the oral liquid of *R. roxburghii* had a Pb repellent effect similar to EDTA, and could reduce the Pb concentration of blood and cure the Pb poisoning without causing trace element disorder [112,113].

### 4.7. Viscera Protection

Human organs are important as well as fragile. Bad habits such as drinking, staying up late, getting angry, and overeating can upset the substance metabolism in the body, disrupt the water-liquid and acid-base balance, and affect the function of organs, thus posing a threat to human health. *R. roxburghii* has rich functional ingredients to protect human organs by regulating metabolic disorders, maintaining water-liquid and acid-base balance. Modern studies show that *R. roxburghii* has a good protective effect on human heart, liver, stomach, spleen, lung, kidney, and other organs. 

#### 4.7.1. Cardioprotection

Flavonoids from *R. roxburghii* have protective effects on cardiovascular and myocardial cells, and also have protective effects on cardiotoxicity caused by chemotherapy drugs. Studies have shown that flavonoids from *R. roxburghii* have a good protective effect on the renal and cardiac function in rats with chronic renal heart syndrome. It can enhance the antioxidant damage ability of the body, improve the parameters of blood rheology, and regulate the excessive stress of endoplasmic reticulum of kidney and heart cells [114]. Molecular studies have shown that flavonoids from *R. roxburghii* play a role in protecting myocardial tissue in the recovery of heart function in rats with chronic heart failure by regulating the expression of integrin β1, FAK and apoptosis-related proteins in the integrin signaling pathway in myocardial tissue of rats with chronic heart failure [115]. In addition, Yuan et al. found that flavonoids from *R. roxburghii* could control the cardiotoxicity caused by adriamycin (DOX) by inhibiting autophagy, and down-regulate the autophagy of cardiomyocytes induced by DOX, thus protecting the cardiotoxicity induced by DOX [116]. 

#### 4.7.2. Gastric Mucosal Protection

Early research found that *R. roxburghii* fruits have effects of strengthening the spleen, aiding digestion, and having the analgesic effect of atropine; the root decoction of *R. roxburghii* has the medicinal value of preventing and treating gastric diseases and protecting gastric mucosa. The *R. roxburghii* root decoction can significantly reduce acute gastric mucosal injury, decrease the rise of lipid peroxide, and improve the activity of SOD without affecting the secretion of gastric acid [117]. In recent years, studies have shown that *R. roxburghii* juice has obvious therapeutic effects on gastric ulcer, and its mechanism may be related to inhibiting gastric acid, pepsin, MDA, and other damage factors, increasing the content of trefoil factor-2 (TFF-2), epidermal growth factor (EGF), and vasoactive substance NO, promoting protective factors such as SOD and prostaglandin E2 (PGE2), producing antioxidant free radicals and anti-inflammatory effects so as to repair and improve gastric mucosa [118,119].

#### 4.7.3. Liver Protection

*R. roxburghii* can improve alcohol-induced drunkenness and liver damage. Studies have shown that *R. roxburghii* has a good anti-alcoholism effect by increasing the activities of liver alcohol dehydrogenase (ADH) and aldehyde dehydrogenase (ALDH) to accelerate ethanol metabolism and reduce the blood ethanol concentration in acute drunken mice. Meanwhile, *R. roxburghii* can also improve the SOD activity and GSH content in the liver of acute hepatic injury mice, enhance the free radical scavenging capacity of the organism, reduce the alanine aminotransferase (ALT) activity, aspartate aminotransferase (AST) activity, and MDA contents in blood, inhibit liver swelling, and play a prominent role in protecting liver [120,121]. Yang et al. studied the anti-alcohol hepatoprotective mechanism of *R. roxburghii* on the gene level [122]. They found that *R. roxburghii* alleviated the overexpression of oxidative stress response genes (Hmox1, Gsta1, Gstm3, Nqo1, Gclc, Vldlr, and Cdkn1a), and promoted the expression of alcohol down-regulated metabolism genes (Angptl8, Slc10a2, Ces3b, Serpina12, C6, and Selenbp2), thereby reducing serum and liver triglyceride levels and effectively resisting chronic alcoholic liver injury. Furthermore, Zhou et al. found that polyphenols from *R. roxburghii* are important active components of *R. roxburghii* playing an anti-alcohol hepatoprotective effect, which can improve ethanol-induced drunkenness and liver injury by inhibiting oxidative stress and lipid peroxidation, and its anti-oxidative stress mechanism may be related to up-regulating the expression of nuclear factor E2-related factor 2 (Nrf2) in liver tissue Nrf2/ARE signaling pathway and activating its downstream antioxidant enzyme heme oxygenase-1 (HO-1) [123].

*R. roxburghii* can alleviate arsenic-induced liver injury. *R. roxburghii* can reduce the accumulation of arsenic in the body, improve the arsenic-induced element metabolism disorder, reverse arsenic-induced weight loss, antagonize arsenic-induced serum and liver oxidative damage and the inhibition of antioxidant enzymes, and effectively inhibit arsenic-induced liver toxicity, thereby alleviating the liver damage caused by coal-fired arsenic [124]. Molecular studies have shown that as can activate the Nrf2/GPX4 signal pathway, increase oxidative stress, and then promote As-induced liver injury of MIHA cells, while *R. roxburghii* can inhibit the Nrf2/GPX4 signal pathway, reduce oxidative stress, and thus reduce As-induced liver injury [125].

#### 4.7.4. Renal Protection

Clinical studies have shown that *R. roxburghii* can resist renal interstitial fibrosis by regulating the expression of “fibroblast growth factor 23 (FGF23)—Klotho protein axis” in patients, which has a good role in delaying the progress of chronic kidney disease, and has a better efficacy and safety for patients with spleen—kidney—deficiency CKD stage 3 [126]. Studies on unilateral ureteral obstruction (UUO) model rats show that *R. roxburghii* can alleviate renal fibrosis and injury in UUO rats by mediating TGF-β/Smads signaling pathway to prevent fibrosis and inhibit oxidative stress [127]. Guo et al. found that both the *R. roxburghii* lyophilized powder and SIRT1 agonist (resveratrol) could reduce the production of lipid peroxides in renal tissue of rats with renal interstitial fibrosis after unilateral ureteral ligation, and increase the contents of antioxidant enzymes, protect the damaged renal tubular epithelial cells, and effectively improve renal fibrosis. Its mechanism might be related to its being rich in vitamin C, SOD, flavonoids, and other antioxidants, which can activate SIRT1 in vivo, thus activating Smad7 to down-regulate the expressions of TGF-β1, TGF-βRI, Smda2, and Smad3 [128].

### 4.8. Other Effects

In addition to the pharmacological activities mentioned above, *R. roxburghii* and its extracts have the effects of improving sleep, anti-inflammatory and analgesic, anti-fatigue, anti-aging, and improving male fertility [65,129]. The side effects of *R. roxburghii* have not been found so far. Moreover, studies have found that *R. roxburghii* flowers, leaves, rhizomes, seeds, and pomace are rich in active substances, which have different edible and medicinal effects and have great development value. For example, *R. roxburghii* leaves are rich in flavonoids, and have strong antioxidant activity, which are the main medicinal ingredients of children’s digestion aperitif Granules [5]. *R. roxburghii* petals are rich in nutrients, especially vitamins, phenols, and anthocyanins, and other antioxidant substances, which have great potential for health food development [17]. *R. roxburghii* seeds can inhibit melanin synthesis by inhibiting tyrosinase activity, so as to play a whitening and skin care role. It can also induce apoptosis of human hepatoma cell HepG2 and effectively prevent and treat tumors [130]. The root decoction of *R. roxburghii* can treat ulcerative colitis, and triterpenoids, ellagic acids, flavonoids and oligosaccharide compounds in its rhizome are the main effective components of its anti-inflammatory effect [131,132]. *R. roxburghii* fruit pomace fermented by pleurotus ostreatus has good bowel soothing and defecating effects [133]. In general, the relevant pharmacological activities and their mechanism and relevant active ingredients of *R. roxburghii* are shown in Table 7.

## 5. Development and Utilization

As the 3G fruit in China, *R. roxburghii* has a unique flavor and homology of medicine and food. Its medicinal and edible value is far higher than that of ordinary fruits. Due to its long-term wild or semi-wild status, it is in short supply in the international market and has gradually become a key industry for local development [1]. Since the 1980s, the new development and use of *R. roxburghii* has rapidly prospered in China. Guizhou, Yunnan, Guangxi, Sichuan, and other provinces have successively established factories and enterprises to develop a large number of *R. roxburghii*-related products. At present, there are a group of leading and demonstrating enterprises in China, which have cultivated a number of well-known and influential brands of *R. roxburghii* products, such as “the King of Cili”, “Golden Cili”, “King of Wild Fruits”, and so on. Brands such as “Panzhou Cili Candied Fruits”, “Golden Cili”, and “Longli Cili” in Guizhou Province have won the authorization of national geographic indication protection products. In addition, under the impetus of the government, *R. roxburghii* processing enterprises cooperate with China Agricultural University, Guizhou University, Taiwan Yilan University, and a number of biological companies to build laboratories and analytical test centers to develop *R. roxburghii* food, skin care products, health products, and drugs, and have successfully developed multiple products. Japanese and French research centers have also begun to import fresh fruits and juice of *R. roxburghii* from China for component test and high-end product development. At present, the developed *R. roxburghii* products in the market include: (1) Food and beverage, such as *R. roxburghii* fresh fruits, *R. roxburghii* beverage, *R. roxburghii* preserved fruits, *R. roxburghii* canned goods, *R. roxburghii* sugar, *R. roxburghii* jam, *R. roxburghii* lamb, *R. roxburghii* buccal tablets, *R. roxburghii* biscuits, *R. roxburghii* wine, *R. roxburghii* tea, *R. roxburghii* jelly, etc. (2) Health care products, such as *R. roxburghii* juice, *R. roxburghii* oral liquid, *R. roxburghii* vinegar, *R. roxburghii* refined powder, *R. roxburghii* freeze-dried powder capsules, etc. (3) Drugs, such as Jincishenjiuzheng Mixture, Kangaifuzheng Capsules, Xuezhiping Capsules, Yishenjianwei Oral Liquid and Cactus Weikang Capsules, etc. (4) Skin care products, such as *R. roxburghii* essence and so on. In addition, *R. roxburghii* medicinal products (e.g., natural VC, *R. roxburghii* SOD capsule) and cosmetics are in the stage of the further research and development [3,134,135].

Although as an important raw material for the domestic food and beverage, alcohol and nutrition, and health products industry, *R. roxburghii* has been developed in a variety of *R. roxburghii* products in China; the common *R. roxburghii* products in life are mainly the low-end products of the industrial chain, such as dried fruit, beverages, preserved fruit cake, *R. roxburghii* fruit juice, and *R. roxburghii* fruit wine, while the market share of the high-end products such as health products, drugs, nutritional supplements, food additives, cosmetics, and super foods is at a low level relatively. Moreover, few products have been researched and developed on the cheap and high-quality natural nutrition resources of *R. roxburghii* flowers, leaves, roots, seeds and pomaces, which have broad development prospect in health care products, tea, and medicine. Although the Chinese *R. roxburghii* industry has developed rapidly in recent years, its large-scale planting, product branding, and the share increase of high-end products remain need to be further strengthened.

## 6. Conclusions and Expectation

In summary, *R. roxburghii* is a potential plant constituent pool with a large number of antioxidants, antibacterials, anti-atherosclerosis, anti-diabetes, and anti-apoptosis compounds. These bioactive plant ingredients allow *R. roxburghii* not only to be developed into a variety of nutritious foods, but also as a unique framework to discover innovative health products, drugs, nutritional supplements, food additives, cosmetics, superfoods, and other high-end products. However, although *R. roxburghii* was included in the 2003 edition of “Quality Standard for Chinese Medicinal Materials and Ethnic Medicinal Materials in Guizhou Province”, the “Standard”only has a simple description of the character description, color reaction, and thin layer identification test of *R. roxburghii*, and lacks specific quantitative indicators. Up until now, there are some studies on the quality control of *R. roxburghii*, but the number of these studies is limited. In terms of qualitative identification, there are some qualitative identifications of fresh fruits, roots, and leaves of *R. roxburghii*. On the other hand, for quantitative determination, there have been some quantitative determinations of vitamin C, phenols, flavonoids, triterpenes and 2 triterpenoid components uscaphic acid and 1-β-hydroxy euscaphic acid in *R. roxburghii* fruits, gallic acid, polyphenols and total flavonoids in *R. roxburghii* leaves, total polyphenols, and ellagic acids in *R. roxburghii* roots, and the determination of four triterpenoids in *R. roxburghii* leaves, roots, stems, and fruits. In terms of quality marker screening, four triterpenoids, including rosolic acid, valeric acid, echinacoside, and rosamultin, were used as quality markers for the quantitative determination of *R. roxburghii* leaves, roots, stems, and fruits. Total polyphenols and ellagic acids were selected as quality markers for *R. roxburghii* roots [136,137,138,139,140,141,142,143]. Overall, although there are some progresses in the research on the quality control of *R. roxburghii*, there are many deficiencies. Firstly, although there are some studies on the quality control of *R. roxburghii* fruits, leaves, and roots, the research into *R. roxburghii* stems and flowers is almost blank. Secondly, there are few studies on quantitative determination and the active components involved are very limited, which are not enough for the reference of screening the quality markers of *R. roxburghii*. Finally, among the existing researches, there is no research to prove that the four triterpenoids of rosa acid, tormentic acid, rosaside, and rosamultin are closely related to any pharmacological activity of *R. roxburghii*; it thus is inappropriate to use them as quality markers. At the same time, the very high content of antioxidant active substance SOD in *R. roxburghii* fruits has not been studied as a quality marker of *R. roxburghii* fruits, which is very regrettable. In view of the current situation of research and development of *R. roxburghii*, we put forward some suggestions from the following aspects:

Firstly, to focus on the qualitative and quantitative studies of organic acids, polyphenols, flavonoid triterpenoids, and polysaccharide components related to the efficacy of *R. roxburghii*, so as to provide the reference for the revision of *R. roxburghii* quality standards.

Secondly, to develop a complex, enhanced nutritional complementary food and drug products based on the nutritional characteristics of *R. roxburghii* and in combination with the nutritional characteristics of other substances. At the same time, to strengthen the research on the pharmacological mechanisms of *R. roxburghii* alleviating or treating corresponding diseases and the development of the related preparations.

Thirdly, research on tannin removal and flavor regulation of *R. roxburghii* fruits and related products should be strengthened to improve and enrich the taste of *R. roxburghii* food and its functional food, so as to improve consumers acceptance and broaden the market of them.

Finally, besides fruits of *R. roxburghii*, more research on its flowers, leaves, roots, seeds, and pomaces should be strengthened to fully develop these cheap and high-quality natural nutrition resources, especially the development of polyphenols, flavonoids and triterpenoids in *R. roxburghii* leaves, which can be used as the source of natural medicines.

## Figures and Tables

**Table 1:** Main nutrients and active components in different parts of *R. roxburghii* [5,13,14,15,16,17,18,19,20].

Nutrients and Active Ingredients		Fruits	Flowers	Leaves
1	Vitamin C (mg/100 g)	276.87~3716.19 (FW)	149.33 (FW)	205.41 (FW)
2	Vitamin E (mg/100 g)	3 (FW)	10.89 (FW)	1.53 (FW)
3	Vitamin P (mg/100 g)	2909 (FW)		
4	Vitamin A (mg/100 g)	0.483 (FW)		
5	Carotene (mg/100 g)	2.9 (FW)		
6	Vitamin B1 (mg/100 g)	0.05 (FW)	0.16 (DW)	
7	Vitamin B2 (mg/100 g)	0.03 (FW)	0.12 (DW)	
8	Vitamin B12 (mg/100 g)		0.07 (DW)	
9	Soluble sugar	4.10%~9.37%	22.71 g/100 g (DW)	2.75%
10	Reducing sugar	3.14%~7.09%	4.77 g/100 g (DW)	1.99%
11	protein	11.62%~26.29%	7.8 g/100 g (DW)	16.04%
12	Total free amino acids (mg/100 g)	1465.8~5755 (FW)	360.51 (DW)	781.63 (DW)
13	Essential amino acid (mg/100 g)	229.3~1292.7 (FW)	39.64 (DW)	93.56 (DW)
14	Important trace element (mg/100 g)	Fe (19.20 FW), Mn (1.77 FW), Zn (1.50 FW), B (1.34 FW), Cu (0.94 FW), P (0.02 FW), K (0.16 FW), Ca (0.04 FW)	Fe (12.03 DW), Mn (7.01 DW), Cu (2.49 DW, Zn (6.89 DW), B (5.42 DW)	Fe (1.58 DW), Mn (0.05 DW), Cu (0.09 DW), Zn (0.22 DW), B (0.05 DW), Mo (0.08 DW)
15	Phenolic acid (mg/100 g)	3356.68 (DW)	1389.61 (DW)	3928.21 (DW)
16	Flavone (mg/100 g)	4956.7 (DW)	1962.55 (DW)	1864.95 (DW)
17	Triterpenoids (mg/100 g)	1055.16 (DW)	804.06 (DW)	1423.64 (DW)
18	Anthocyanin (mg/100 g)		179.97 (DW)	
19	Total ellagic acid (mg/100 g)	4936.37 (DW)		19,708.54 (DW)
20	SOD (U/g)	22,000 (DW)	2500 (DW)	600 (DW)

**Table 2:** Comparation of important vitamin and mineral contents of *R. roxburghii* fresh fruits and other common fresh fruits (calculated per 100 g edible part) [2,22].

Fruit	*R. roxburghii*	Apple	Banana	Lemon	Lychee	Strawberry	Ficus	Pineapple	Kiwifruit	Mulbrry
Edible part (%)	100	85	59	66	73	97	100	68	83	100
Moisture (g)	81.00	86.10	75.80	91.00	81.90	91.30	81.3	88.40	83.40	82.80
Energy (kcal)	63.00	53.00	93.40	37.00	71.00	32.20	65.0	43.60	61.20	57.20
Vitamin A (μg)	483.00	4.00	5.00	150.00	1.00	2.50	2.50	20.00	11.00	2.50
Vitamin B1 (mg)	0.05	0.02	0.02	0.05	0.10	0.02	0.03	0.04	0.05	0.02
Vitamin B2 (mg)	0.03	0.02	0.04	0.02	0.04	0.03	0.02	0.02	0.02	0.06
Vitamin C (mg)	2586.00	3.00	8.00	22.00	41.00	47.00	2.00	18.00	62.00	-
Vitamin E (mg)	3.00	0.43	0.24	1.14	-	0.71	1.82	-	2.43	9.87
Carotene (μg)	2900	20.00	60.00	-	10.00	30.00	-	20.00	130	30.00
Fe (mg)	19.20	0.30	0.40	0.80	0.40	1.80	0.10	0.60	1.20	0.40
Zn (mg)	1.50	0.04	0.18	0.65	0.17	0.14	1.42	0.14	0.57	0.26
Cu (mg)	0.94	0.07	0.14	0.14	0.16	0.04	0.01	0.07	1.87	0.07
Mn (mg)	1.77	0.03	0.65	0.05	0.09	0.49	0.17	1.04	0.73	0.28

**Table 3:** Components and bioactivities of *R. roxburghii* polysaccharide components.

Time	Components	Molecular Weight	Components (Molar Ratio/Molar Percentages)	Bioactivities	References
2018	PR-1	7.4~6.2 × 10^3^ KDa	Man (2.10%), Ribose (0.54%), Rha (2.10%), GluH (0.26%), GlcA (1.50%), GalA (22.70%), Glc (24.0%), Gal (26.40%), Ara (19.60%), Fuc (0.89%)	Antioxidant and strong α-D-glucosidase inhibitory activity	[46]
2018	PR-2	106.6~559.8 KDa	Man (3.2%), Ribose (0.8%), Rha (3.2%), GluA (2.2%), GalA (34.8%), Glc (12.1%), Gal (24.0%), Ara (19.4%)	Antioxidant and strong α-D-glucosidase inhibitory activity	[46]
2018 2020	RTFP	--	Ara (33.80%), Gal (37.30%), Glc (20.70%), Man (1.74%), Xyl (3.43%), Fu (2.95%)	α-Amylase and α-glucosidase inhibitory capacities; Ameliorating hyperglycemia and hyperlipidemia; reversing diabetes-induced gut disorder	[48,49]
2018	RTFP-3	67.2 KDa	Ara (37.20%), Gal (34.14%), Glc (10.02%), Man (0.15%), Xy l (0.17%), Fu (18.30%),	α-Glucosidase inhibitory activity, attenuating oxidative stress and antimicrobial ability	[47,54,57]
2019	RSPs-40	228.3 kDa	Ara:Gal:Glc:Fru:GalA = 0.24:0.37:3.22:0.27:1.44	Antioxidant and stronger α-glucosidase inhibitory activity than acarbose.	[50]
2019	RSPs-60	124.14 kDa	Ara:Gal:Glc:GalA = 1.58:2.06:2.37:1.69	Antioxidant and stronger α-glucosidase inhibitory activity than acarbose.	[50]
2019	*RT*P1-1	97.58 kDa	Man:AsA:Rha:GlcA:GalA:Glc:Gal:Ara:Xyl = 2.88:1.39:2.83:1.00:69.11:3.04:2.52:3.41	Antioxidant and anti-aging	[51]
2021	RTFP-30	515.1 kDa	Fuc:Rha:Ara:Gal:Glc:Xyl:Man:GalA:GluA = 2.56:3.22: 24.55:30.81:29.24:1.17:1.88:4.79:1.83	Antioxidant and prevention of obesity and hypercholesterolemia	[52]
2021	RTFP-50	517.3 kDa	Fuc:Rha:Ara:Gal:Glc:Xyl:GalA:GluA = 2.73:4.85:15.38:26.35:10.52:2.06:37.17:0.94	Antioxidant and prevention of obesity and hypercholesterolemia	[52]
2021	RTFP-80	176.5 kDa	Fuc:Rha:Ara:Gal:Glc:Xyl:GalA:GluA = 2.21:5.16:18.00:28.21:11.20:0.57:33.46:1.19	Antioxidant and strong α-glucosidase inhibitory activity	[52]
2005	R*RT*P-1	3200 kDa	Rha:Ara:Unknown monose:Xyl:Man:Gal:Clu = 1.00:16.75:13.37:5.86:11.49:22.73:7.80	Remarkable protective effect on neural stem cells damage induced by Na_2_S_2_O_3_.	[55]
2022	RRTFP-2	74.33 kDa	Rha:Ara:Man:Glc:Gal:GluA:GalA = 1.1:6.5:1.1:1.2:1.2:16.1	NGF-like neurotrophic activity.	[56]
2022	RTFP-1	128.7 kDa	Ara (34.84%), Gal (40.59%), Glc (12.11%), Man (5.06%), Xyl (3.39%), and Fuc (4.01%)	Chemopreventive and antitumor agent	[53]

**Table 4:** Components of flavonoid and phenolic acid in fruits, petals, and leaves of *R. roxburghii* [15].

Components		Fruits	Petals	Leaves
		(mg/100 g DW)	(mg/100 g DW)	(mg/100 g DW)
Flavonoid	Catechin	1114.18	720.70	867.41
	Epicatechin	227.60	284.00	194.95
	Rutin	681.27	88.21	88.37
	Quercetin	644.40	49.64	9.85
	Quercitrin	420.82	159.02	65.03
	Isoquercitrin	269.07	50.10	138.38
	Luteolin	411.73	54.91	44.72
	Myricetin	614.65	186.88	289.47
	Kaempferol	421.91	64.65	36.43
	Proanthocyanidins	14.25	95.44	4.98
	Apigenin	27.48	12.03	7.84
	Naringenin	49.53	53.22	9.67
	Naringin	59.81	143.75	107.85
	Total content of flavonoid	4956.7	1962.55	1864.95
Phenolic acid	Gallic acid	80.05	92.46	138.06
	Protocatechuic acid	135.42	144.93	200.63
	Caffeic acid	70.79	313.59	326.69
	Syringic acid	30.47	11.38	267.50
	P-coumaric acid	62.26	41.71	15.09
	Chlorogenic acid	131.90	45.63	1363.17
	Ferulic acid	49.88	88.64	50.45
	Vanillic acid	110.27	354.52	821.39
	Rosmarinic acid	60.66	163.21	225.54
	Tannic acid	2624.98	133.54	519.68
	Total content of flavonoid	3356.38	1389.61	3928.8

**Table 5:** Components and contents of triterpenoids acid in fruits, petals, and leaves of *R. roxburghii* [15].

Components	Fruits	Petals	Leaves
	(mg/100 g DW)	(mg/100 g DW)	(mg/100 g DW)
Echinacoside	441.16	133.72	600.41
Roseoside	69.37	45.15	238.81
Rosolic acid	681.27	614.7	566.13
Ursolic acid	530.81	10.49	18.29
Total content	1055.16	804.06	1423.62

**Table 6:** Comparison of constituents and in vitro antioxidant activities of fresh and dried. *R. roxburghii* fruits [31].

Constituents		Fresh Fruits	Dried Fruits
Amino acids		21.34%	34.29%
Acylamide		0%	11.29%
Flavonoids		35%	22.13%
Main component of flavonoids		Catechin or its isomer (13.25%), procyanidin B1 or its isomer (8.96%)	Catechin or its isomer (10.32%), procyanidin B1 or its isomer (4.44%)
Organic acids		60.37%	52.81%
Main component of organic acids		L-ascorbic acid (30.8%)	L-ascorbic acid (25.28%)
Phenols		3.88%	19.10%
Main component of phenols		Ellagic acid (1.31%)	Ellagic acid (4.84%) and gallic acid (4.54%)
Terpenoids		16.56%	13.3%
DPPH radical scavenging activity (mg AAE/g)	DPPH	2.92	5.05
	IC_50_ (mg/mL)	4.21	3.69
FRAP value (mmol Fe^2+^/100 g)	FRAP	110.62	187.3

Abbreviations: DPPH·, 1,1-diphenyl-2-picrylhydrazyl radical; FRAP, ferric reducing antioxidant power; the DPPH radical scavenging activity of the sample was expressed as mg of L-ascorbic acid equivalents (AAE) per gram of dried sample (mg AAE/g); the FRAP value was calculated as millimoles of Fe^2+^ equivalents per 100 g of sample (mmol Fe^2+^ equiv/100 g) based on a calibration curve plotted using FeSO_4_·7H_2_O as the standard curve.

**Table 7:** Pharmacological activities of *R. roxburghii* and their related mechanisms and active components.

No.	Functions	Mechanisms	Active Components	References
1	Antioxidant activity	Scavenging free radicals, regulating the activity of related enzymes, chelating metal ions (Fe3+), regulating the level of Na+, K+, regulating the expression of related genes, increasing the content of beneficial substances, and reducing the content of harmful substances.	Polysaccharides, polyphenols, flavonoids, vitamin C, triterpenesand and SOD	[9,15,19,31,46,50,51,52,63,64,65,66,67,68,69,70,71]
2	Immune regulation	Reducing the expression of immune inflammatory factors, increasing the content and phagocytosis of macrophages, improving the ability to resist oxidative stress, regulating the immune microenvironment, and enhancing immune function.	Polysaccharide, triterpenoid	[72,73,74,75]
3	Reducing blood glucose	Inhibiting the activity of related enzymes to decrease the absorption rate of blood glucose; regulating the expression of protease through multiplying cellular signaling pathways.	Polysaccharides, polyphenols, flavonoids and triterpenes.	[46,47,48,49,50,52,54,62,78,79,80,81]
4	Reducing blood lipid	Improving the activity of related enzymes, regulating the expression of related proteases and binding cholesterol, bile acids and the fat of the body.	Polysaccharide, phenolic acids	[52,82,83,84]
5	Modulating intestinal flora and lipid metabolism	Increasing the abundance of beneficial groups and their related metabolites and SCFAs in intestinal tract; down-regulating the key enzymes related to fatty acid synthesis in liver lipid metabolism; regulating the expression of related factors; promoting the transformation of adipose tissue macrophages from M1 phenotype to M2 phenotype. Improving the level of lipid decomposition and reducing the accumulation of fat in the body.	Polysaccharides, polyphenols, flavonoids, and vitamin C	[49,85,86,87]
6	Anti-atherosclerosis	Reducing the contents of HDL, TC and TG, inhibiting ox-VLDL and foam-stimulating cell, improving SOD activity, regulating lipid metabolism, reducing lipid and oxidative damage, and preventing plaque formation.	Closely related to vitamin C	[89,90,91,92]
7	Antitumor	Controlling the expression of related genes and molecules, blocking the synthesis of carcinogens, inducing the apoptosis of cancer cells, and inhibiting the proliferation of tumor cells; down-regulating the expression of bad mRNA and inducing cell differentiation.	Polysaccharides, triterpenes	[93,94,95,96,97,98,99,100]
8	Anti-radiation	Regulating related genes and signaling pathways, reducing ROS production and cells’ apoptosis.	Flavonoids	[101,102,103]
9	Heavy metal detoxification	Promoting the excretion of heavy metals, supplementing the body trace elements, reducing peroxide damage, improving SOD activity, and enhancing body immunity.	Polysaccharide, vitamin C, SOD, and trace elements	[105,106,107,108,109,110,111,112]
10	Cardioprotection	Enhancing the ability of antioxidant damage, improving blood rheology, regulating the excessive stress of endoplasmic reticulum; regulating related signaling pathways and key proteins, and down-regulating cardiac autophagy.	Flavonoids	[113,114,115]
11	Gastric mucosal protection	Inhibiting injury factors, increasing the content of protective factors and vasoactive substances, and promoting antioxidant and anti-inflammatory effects.	Antioxidant and anti-inflammatory related active ingredients	[116,117,118]
12	Reducing alcoholic liver injury	Regulating related gene expression and protease activity, accelerating ethanol metabolism, reducing oxidative stress, and reducing the level of harmful substances in liver and serum.	Polyphenols	[119,120,121,122]
13	Alleviating coal-fired arsenic poisoning liver injury.	Reducing the accumulation of arsenic in the body, inhibiting the damage-related signaling pathways, improving the disorder of elemental metabolism and weight loss caused by arsenic poisoning, and reducing oxidative stress injury.	Active substances related to heavy metal detoxification	[123,124]
14	Renal protective function	Reducing oxidative stress injury, regulating related signaling pathways and proteases, and resisting renal interstitial fibrosis.	Flavonoids, vitamin C and SOD	[125,126,127]

## Data Availability

Data is contained within the article.
